# For Residents, by Residents: Developing a Physician Handoff Tool at a University Affiliated Community Hospital

**DOI:** 10.7759/cureus.18352

**Published:** 2021-09-28

**Authors:** Lu Chen, Uta S Guo, Siddharth Bhesania, Hardikkumar Shah, Emdad Ali, Parag Mehta

**Affiliations:** 1 Department of Cardiology, State University of New York Downstate Medical Center, Brooklyn, USA; 2 Department of Family Medicine and Community Health, Icahn School of Medicine at Mount Sinai, New York, USA; 3 Internal Medicine, Overlook Medical Center, Summit, USA; 4 Internal Medicine, NewYork-Presbyterian Brooklyn Methodist Hospital, Brooklyn, USA; 5 Gastroenterology and Hepatology, Saint Joseph's University Medical Center, Paterson, USA; 6 Internal Medicine, OhioHealth Physician Group, Columbus, USA

**Keywords:** physician satisfaction, patient safety, electronic medical records, standardized handoff, graduate medical education (gme)

## Abstract

The 80-hour per week work limit resulted in an increased number of patient handoffs. A satisfactory handoff system should optimize the exchange of vital patient information while concisely minimizing error. This project describes our experience and lessons learned in successfully developing and implementing an Electronic Health Record (EHR)-integrated handoff system based on the I-PASS model. The handoff system, termed Physician Handoff, was refined through end-user feedback. End-users were evaluated on the quality of handoff in the following categories: Illness Severity, Patient Summary, Action List, and Situational Awareness. Resulting survey showed high adoption and satisfaction rate with Physician Handoff. Success can be attributed to interdepartmental collaboration, credentialing the users, and recognizing the importance of end-user feedback.

## Introduction

The devil is in the details. No matter how much the importance of communication is emphasized in our training; errors in communication is one of the leading causes of sentinel events reported [1]. This is critical in graduate medical education. Since the 80 duty-hour per week restriction was put into effect [2], there is an increased number of handoffs performed by resident physicians [3]. Compared to 2003, there has been a 40% increase in handoffs performed by physicians. On average, a resident physician may be involved in over 300 handoffs during a month-long rotation. A patient would be handed off 15 times over a five-day hospital course [4]. Unfortunately, the communication during handoff can overly rely on written handoffs, and the effectiveness of communication is often overestimated [5,6]. Boosted confidence level and increased numbers of handoffs can make handoffs more vulnerable to errors.

Previous studies found that four out of five handoff sheets contain at least one error, with the most common error being medication omission [7]. Additionally, half of the patient handoff documents become inaccurate or outdated within six hours on an average night shift, mostly secondary to medication changes. By the following morning, merely 40% of handoff documents were still current [8]. A few studies have noted an association between handoff quality and patient outcomes. In a critical care setting, more structured handoffs were associated with a decreased number of non-routine clinical events [9]. In the surgical arena, the transition from written to electronic handoff resulted in a reduction in length of stay [10]. Another study described residents inappropriately performing resuscitation on multiple patients due to discrepancies found on handoff sheets. There were also patients who were supposed to be resuscitated but were denied resuscitations due to handoff errors [11].

In order to minimize errors and prevent adverse clinical events, it is necessary that the information on handoff documents is accurate, concise, and complete. Ideally, a handoff system should be fully integrated into the Electronic Health Records (EHR). When available, clinical information in the handoff system should be generated and updated automatically to minimize manual input and errors. A previous study has shown that automatically generated handoff increased the completeness of handoff, minimized errors, and streamlined physician workflow [12,13]. Using EHR-integrated handoffs is associated with a reduction in length of stay for the patients and improvement in provider satisfaction [14]. Early adapters of EHR-integrated handoff found it consistent and generalizable across different study sites [15]. To improve patient safety and comply with Accreditation Council for Graduate Medical Education (ACGME) regulation, our hospital has developed and implemented an EHR-integrated handoff system that provides clear, accurate, and concise patient handoff sheets. The purpose of this paper is to describe our experience and the lessons we have learned in designing a satisfactory handoff system for resident physicians in our hospital.

## Technical report

Previous versions of our handoffs

**Figure 1:**
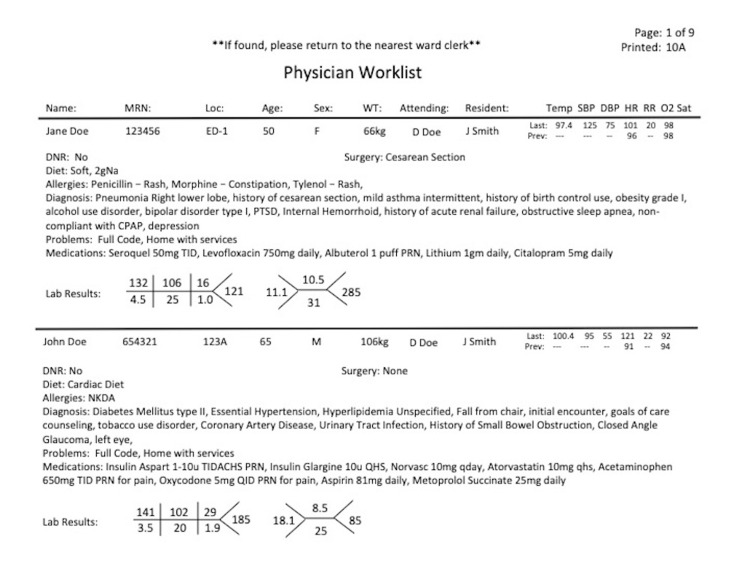
Sample of Printed Physician Worklist MRN: medical records number; Loc: location; WT: weight; Temp: temperature; SBP: systolic blood pressure; DBP: diastolic blood pressure; HR: heart rate; RR: respiratory rate; O2 Sat: oxygen saturation; ED: emergency department; kg: kilogram; Prev: previous; DNR: do not resuscitate; 2gNa: 2 gram sodium; PTSD: post-traumatic stress disorder; CPAP: continuous positive airway pressure; TID: 3 times per day; PRN: as needed; NKDA: no known drug allergy; TIDACHS: 3x per day, before meals and bedtime; u: units; qday: once per day; qhs: nightly; QID: 4x per day

Patient handoff was previously conducted verbally and supplemented with either written index cards or manually inputted handoff into word-processing software per provider preference. Since the implementation of EHR (Cerner Corporation, Kansas City, MO) at our hospital in 2011, efforts were made to integrate electronic handoff into the EHR. The Information Technology (IT) department and administration integrated Physician Worklist, an existing built-in handoff program into our EHR system (Figure 1). Physician Worklist was designed to be comprehensive and automated, which included information such as code status, vital signs, diet, allergy, diagnosis, active problems, medications, and laboratory results. Unfortunately, Physician Worklist did not include physician assessment, plan of care, and patient disposition. The volume of information limited number of patients per printed page, resulting in 40-60-page sign out packets for the night team residents. End-user survey following implementation showed poor adoption rate and low satisfaction with Physician Worklist.

Physician handoff

A decision was made to implement a more user-friendly handoff system. Two senior resident physicians were elected to lead the project. They were designated as resident liaisons between the Internal Medicine (IM) leadership, the IT department, and fellow resident physicians. The newly developed handoff system was structured according to a published handoff system, which has been linked to reduction in medical errors and adverse events [16-18]. The published handoff system is known for its mnemonic I-PASS (Illness Severity, Patient Summary, Action List, Situational Awareness, and Synthesis by Receiver). We adapted the I-PASS model and termed our new handoff Physician Handoff.

**Figure 2:**
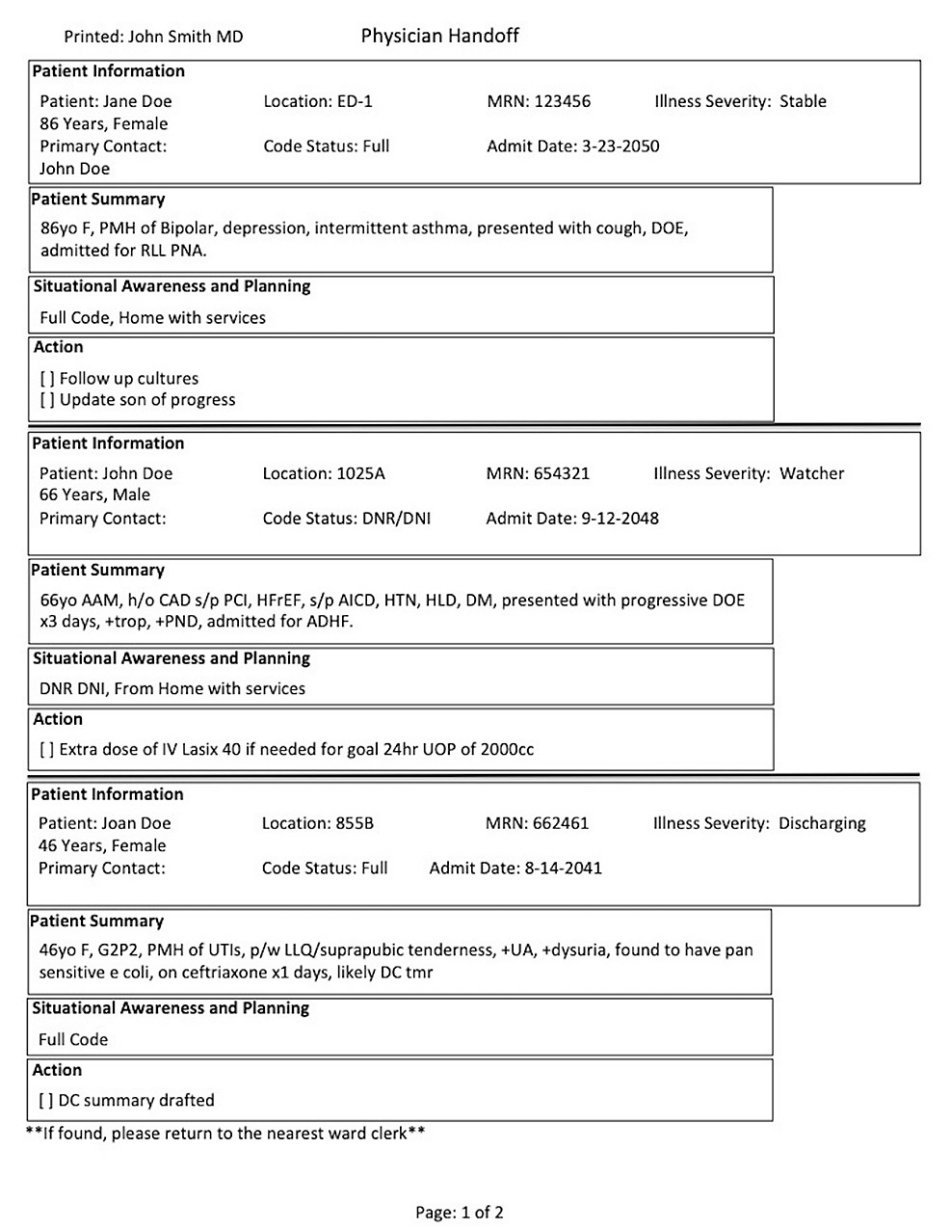
Sample of Printed Physician Handoff ED: emergency department; MRN: medical records number; F: female; PMH: past medical history; DOE: dyspnea on exertion; RLL: right lower lobe; PNA: pneumonia; DNR/DNI: do not resuscitate/do not intubate; AAM: African American male; CAD: coronary artery disease; PCI: percutaneous coronary intervention; HFrEF: heart failure reduced ejection fraction; AICD: automatic implantable cardioverter defibrillator; HTN: hypertension; HLD: hyperlipidemia; DM: diabetes mellitus; DOE: dyspnea on exertion; PND: paroxysmal nocturnal dyspnea; ADHF: acute decompensated heart failure; IV: intravenous; UOP: urine output; G2P2: Gravidity 2 Parity 2; UTI: urinary tract infection; LLQ: left lower quadrant; UA: urinalysis; DC: discharge

The patient summary section in Physician Handoff was limited to 500 characters, allowed more patients to fit per printed page. Action list allowed providers to create an individualized task list. Physician Handoff auto-populated Code Status, Diagnosis, Physician Contact, and Admission Date, further minimizing input errors (Figure 2). Laboratory finding and medications were omitted from the printed version, but can be easily accessed on computerized version. Handoff can be reviewed, verified, and modified by any member of the care team at any secured workstation with EHR access, ensuring secured and real-time patient information sharing.

Implementation of Physician Handoff required multiple rounds of fine-tuning due to the need of different departments. Following the development of Physician Handoff, all residents were trained and credentialed in the new handoff system. Residents were evaluated on both written and verbal handoffs. Anonymous surveys following implementation showed a better adoption rate and higher satisfaction rate among resident physicians.

## Discussion

Developing, implementing, and evaluating an efficient EHR-integrated handoff system was a momentous undertaking for all individuals involved (IM leadership, IT department, and the resident physicians). Valuable lessons were learned during this process: 1) interdepartmental collaboration; 2) credential the users, and most importantly, 3) value end-user’s opinions and workflows. By applying the knowledge gained from these lessons, we were able to develop and implement Physician Handoff, a widely accepted system that brought many unforeseen benefits.

Over the span of three years, two EHR-integrated handoff systems were introduced in our hospital to replace manually inputted handoff. Physician Worklist focused on completeness but failed to incorporate end-user feedback. In contrast, Physician Handoff was designed by resident physicians with an emphasis on end-user experience. Differences among the three handoff systems are summarized in Table 1.

**Table 1:** Handoffs At a Glance: Comparison of Manual Handoff, Physician Worklist, and Physician Handoff IM: Internal Medicine; IT: Information Technology; EHR: Electronic Health Record; HIPAA: Health Insurance Portability and Accountability Act

	Manual Handoff	Physician Worklist	Physician Handoff
Designed by	Residents	IT & IM Leaders	IT, IM Leaders, Resident
Resident Feedback	Incorporated	Not Incorporated	Incorporated
EHR Integration	No	Yes	Yes
Availability of Access	Single Workstation	Hospital Wide	Hospital Wide
HIPAA Compliance	No	Yes	Yes
Content	Varies	Comprehensive	Concise
Free Text Option	Yes	Limited	Yes
Automation	No	Yes	Yes
Formalized Training	No	No	Yes

Lesson 1: Interdepartmental collaboration

The successful development of Physician Handoff can be attributed to the perseverance and collaboration of the multidisciplinary team. Through multiple setbacks, we enacted the Plan-Do-Study-Act (PDSA) workflow, which included: working closely with the IT department and EHR vendor, assigning senior residents as liaisons, and holding discussions with IM leadership.

IM Departmental leadership served as an oversight to the project but allowed resident liaisons and IT department to refine the details. This style of leadership maximized incorporation of residents’ ideas within a set framework. Feedbacks from numerous attending physicians were also conveyed through the department leadership. Resident liaisons serve the crucial role of learning and summarizing the opinion of resident physicians. They voiced the concerns of residents in a concise, and non-confrontational manner. The suggestions for modifications from any party were only incorporated into the handoff design upon agreement by all three parties (IM, IT, and liaisons). Our IT department provided unique insight from the design aspect and troubleshot various technical challenges in incorporating our new handoff system. Their innovative designs were a novice to many clinicians and made them invaluable members of the design team.

Lesson 2: Credential of the users

Once Physician Handoff debuted, the resident liaisons and the IM leadership were certified as super users of the new system. The super users worked together and designed a complete but concise training course for the remaining users to allow a seamless transition. A formal training ensured that the end-users had a uniform and adequate understanding of not only the I-PASS handoff model but also Physician Handoff program and the services it offered. Following the credentialing course, resident liaisons personally observed and assessed individual handoffs based on all categories of the I-PASS model. The success was contributed to properly credentialing of the end-users and perseverance of the liaisons.

Lesson 3: Value end-user’s opinions and workflows

The development of Physician Worklist neither include end-user feedback, nor did it provide the components essential to resident physicians (plan of care, disposition, and task list). Our study is consistent with the previous studies that have demonstrated the importance of collaborating with end-users in both the development and implementation process of handoff systems [19-22]. Building on user feedback, the inclusion of the “Comment” and “Actions” sections allowed for a personalized aspect of health care to be communicated between providers. Physician Handoff displayed information vital to providers caring for the patient. Due to its conciseness, more patients were listed per page. Because the opinions of the end-users were addressed and incorporated, Physician Handoff was better adapted with a high satisfaction rate.

Physician handoff: A step in the right direction

Physician Handoff is a concise and EHR-integrated handoff system that maximized automation while allowed freedom of manual input. By automatically updating code status and prominently displaying it on the handoff sheet, this build-in feature minimizes errors associated with manual input and can potentially limit inappropriate resuscitations. Physicians identified illness severity on handoff helped in better allocation of resources for both nursing and supporting staff. Additional advantages of an EHR-integrated handoff system include enhancing compliance with Health Insurance Portability and Accountability Act (HIPAA) and providing accurate information transfer. EHR-integrated handoff tools can ensure a secure hospital-wide exchange of patient information, preserve the continuity of care, and prevent potential adverse events in our patients. The effect of this implementation on our patient care is yet to be determined.

Our experience with implementing a standardized and EHR-integrated handoff system echoed the experience of many previous studies. Lessons we have learned independently mirrored the recommendation of a recent literature review [23]. In our experience, collaboration, improvisation, adequate credentialing, and the inclusion of end-users in the design and revision efforts were the roots of our success.

## Conclusions

In an effort to improve patient safety we developed a satisfying and widely accepted handoff system. Developing an EHR-integrated handoff tool was a monumental undertaking, requiring coordination between IM departmental leadership, IT Department, and end-users. Our mission was to minimize handoff errors in Physician Handoff by automating objective data and limit manual input. We attributed that success to collaborating, improvising, credentialing the users, and recognizing the importance of end-user feedback.

## References

[1] Dingley C, Daugherty K, Derieg MK, Persing R (2008). Improving patient safety through provider communication strategy enhancements. Advances in Patient Safety: New Directions and Alternative Approaches.

[2] Drolet BC, Spalluto LB, Fischer SA (2010). Residents' perspectives on ACGME regulation of supervision and duty hours--a national survey. N Engl J Med.

[3] DeRienzo CM, Frush K, Barfield ME (2012). Handoffs in the era of duty hours reform: a focused review and strategy to address changes in the Accreditation Council for Graduate Medical Education Common Program Requirements. Acad Med.

[4] Okie S (2007). An elusive balance--residents' work hours and the continuity of care. N Engl J Med.

[5] Chang VY, Arora VM, Lev-Ari S, D'Arcy M, Keysar B (2010). Interns overestimate the effectiveness of their hand-off communication. Pediatrics.

[6] Blondon K, Zotto MD, Rochat J, Nendaz MR, Lovis C (2018). A simulation study on handoffs and cross-coverage: results of an error analysis. AMIA Annu Symp Proc.

[7] Arora V, Kao J, Lovinger D, Seiden SC, Meltzer D (2007). Medication discrepancies in resident sign-outs and their potential to harm. J Gen Intern Med.

[8] Rosenbluth G, Jacolbia R, Milev D, Auerbach AD (2016). Half-life of a printed handoff document. BMJ Qual Saf.

[9] Nanchal R, Aebly B, Graves G (2017). Controlled trial to improve resident sign-out in a medical intensive care unit. BMJ Qual Saf.

[10] Ryan S, O'Riordan JM, Tierney S, Conlon KC, Ridgway PF (2011). Impact of a new electronic handover system in surgery. Int J Surg.

[11] Vidyarthi AR, Arora V, Schnipper JL, Wall SD, Wachter RM (2006). Managing discontinuity in academic medical centers: strategies for a safe and effective resident sign-out. J Hosp Med.

[12] Tisdale RL, Eggers Z, Shieh L (2018). EMR-based handoff tool improves completeness of internal medicine residents' handoffs. BMJ Open Qual.

[13] Bernstein JA, Imler DL, Sharek P, Longhurst CA (2010). Improved physician work flow after integrating sign-out notes into the electronic medical record. Jt Comm J Qual Patient Saf Jt Comm Resour.

[14] Palma JP, Sharek PJ, Longhurst CA (2011). Impact of electronic medical record integration of a handoff tool on sign-out in a newborn intensive care unit. J Perinatol.

[15] Stein DM, Vawdrey DK, Stetson PD, Bakken S (2010). An analysis of team checklists in physician signout notes. AMIA Annu Symp Proc.

[16] Starmer AJ, Spector ND, Srivastava R, Allen AD, Landrigan CP, Sectish TC, the I-PASS Study Group (2012). I-pass, a mnemonic to standardize verbal handoffs. Pediatrics.

[17] Starmer AJ, Sectish TC, Simon DW (2013). Rates of medical errors and preventable adverse events among hospitalized children following implementation of a resident handoff bundle. JAMA.

[18] Starmer AJ, Spector ND, Srivastava R (2014). Changes in medical errors after implementation of a handoff program. N Engl J Med.

[19] Graham KL, Marcantonio ER, Huang GC, Yang J, Davis RB, Smith CC (2013). Effect of a systems intervention on the quality and safety of patient handoffs in an internal medicine residency program. J Gen Intern Med.

[20] Patel VP, Raptis D, Christofi T (2009). Development of electronic software for the management of trauma patients on the orthopaedic unit. Injury.

[21] Rao BS, Lowe GO, Hughes AJ (2012). Reduced emergency calls and improved weekend discharge after introduction of a new electronic handover system. Med J Aust.

[22] Wohlauer MV, Rove KO, Pshak TJ (2012). The computerized rounding report: implementation of a model system to support transitions of care. J Surg Res.

[23] Davis J, Riesenberg LA, Mardis M, Donnelly J, Benningfield B, Youngstrom M, Vetter I (2015). Evaluating outcomes of electronic tools supporting physician shift-to-shift handoffs: a systematic review. J Grad Med Educ.

